# Evaluation of reference genes for real-time PCR studies of Brazilian Somalis sheep infected by gastrointestinal nematodes

**DOI:** 10.1590/S1415-47572010000300018

**Published:** 2010-09-01

**Authors:** Lilian Giotto Zaros, Luiz Lehmann Coutinho, Lúcia Helena Sider, Henrique Rocha de Medeiros, Maria Rosalba Moreira das Neves, Camila Loures Benvenuti, Andrine Maria do Carmo Navarro, Luiz da Silva Vieira

**Affiliations:** 1 Universidade Federal do Rio Grande do Norte, Natal, RNBrazil; 2 Escola Superior de Agricultura Luiz de Queiroz, Universidade de São Paulo, Piracicaba, SPBrazil; 3 Embrapa Caprinos e Ovinos, Sobral, CEBrazil; 4 Universidade Estadual Vale do Acaraú, Sobral, CEBrazil

**Keywords:** control genes, gastrointestinal nematodes, ovine, real-time RT-PCR

## Abstract

Precise normalization with reference genes is necessary, in order to obtain reliable relative expression data in response to gastrointestinal nematode infection. By using sheep from temperate regions as models, three reference genes, viz., ribosomal protein LO (RPLO), glyceraldehyde 3-phosphate dehydrogenase (GAPDH) and succinate dehydrogenase complex subunit A (SDHA), were investigated in the abomasum, abomasal lymph nodes and small intestine of Brazilian Somalis sheep, either resistant or susceptible to gastrointestinal nematodes infections. Real time PCR was carried out by using SYBR Green I dye, and gene stability was tested by geNorm. RPLO was an ideal reference gene, since its expression was constant across treatments, presented lower variation, and was ranked as the most stable in abomasum and lymph node tissues. On the other hand, SDHA was the most stable in the small intestine followed by RPLO and GAPDH. These findings demonstrate the importance of correctly choosing reference genes prior to relative quantification. In addition, we determined that reference genes used in sheep from temperate regions, when properly tested, can be applied in animals from tropical regions such as the Brazilian Somalis sheep.

Gastrointestinal nematode parasitism causes severe losses in sheep production (Ingham *et al.*, 2008). Animals respond to this infection by activating the proliferation of T Helper (T_H_) which secretes cytokines related to resistance and susceptibility to gastrointestinal nematode infections (Zaros *et al.*, 2007). The study of these and other molecules is important for understanding the mechanisms involved in immune response to gastrointestinal nematode infections.

Several methods and techniques can be used for measuring mRNA levels, such as real-time PCR (RT-PCR) technology, which provides sensitivity and accuracy (Garcia-Crespo *et al.*, 2005). Nevertheless, several variables need to be controlled in gene expression analysis, such as the amount of starting material, RNA integrity and enzymatic efficiency, as well as differences between tissues and cells in overall transcription activities (Vandesompele *et al.*, 2002; Andersen *et al.*, 2004). These controls can be obtained by using internal control genes, called reference genes or housekeeping genes, whose expression should not vary among tissues or cells under investigation (Andersen *et al.*, 2004). An ideal reference gene is universally valid, with a constant expression level across all tissue samples, cells, experimental treatments and designs (Vandesompele *et al.*, 2002).

The identification of ideal reference genes in studies with ruminants, such as sheep and cattle, has been the topic of several publications from Australia, New Zealand, Canada and the United States (Cao *et al.*, 2006; Jasmer *et al.*, 2007; Smeed *et al.*, 2007), yet little has been reported on their use in tropical cattle infected by parasites (Zaros *et al.*, 2007; Bricarello *et al.*, 2008; Regitano *et al.*, 2008) and none in sheep. Thus the importance of analyzing whether previously reported reference genes, extensively used in studies of animals from temperate regions, are applicable to sheep raised in the tropics.

The aim of this work was to evaluate the stability of three reference genes – ribosomal protein LO (RPLO), glyceraldehyde 3-phosphate dehydrogenase (GAPDH) and succinate dehydrogenase complex subunit A (SDHA) - in Brazilian Somalis sheep resistant and susceptible to gastrointestinal nematode infections.

A herd of three to four-month-old Brazilian Somalis sheep were raised on native caatinga pasture at “Embrapa Caprinos e Ovinos” in Sobral, Ceará, in north-eastern Brazil (3°41'32” S e 40°20'53” W). The animals were kept for 98 days in pasture naturally infested with nematode larvae and without anthelmintic treatment. Individual body weight, blood and fecal samples were collected weekly. Based on mean fecal egg counts (FEC) over the 98 days, the eight most resistant animals (*i.e.* those with the lowest mean FEC) were classified as the resistant group, whereas the eight most susceptible (*i.e.* those with the highest mean FEC) were classified as the susceptible group. Tissues samples from animals in both groups were collected after slaughter.

Tissue samples from the abomasum, abomasal lymph nodes and small intestine were collected immediately after slaughter, frozen in liquid nitrogen, and then transported to the laboratory, where they were maintained at -80 °C. Total RNA was extracted according to Chomczynski and Sacchi (1987), using Trizol Reagent (Invitrogen Co., Carlsbad, CA, USA). RNA concentration was assessed by spectrophotometry at OD 260 nm, purity checked by the OD 260/280 ratio, and integrity verified by denaturing agarose-gel electrophoresis. cDNA was reverse transcribed from 5.0 μg of total RNA by using an oligo(dT) primer and Superscript II (Invitrogen Co, Carlsbad, CA, USA), according to manufacturer's instructions.

Three reference genes, RPLO, GAPDH and SDHA, were tested. Primer sequences were obtained from Cao *et al.* (2006) for RPLO and Smeed *et al.* (2007) for GAPDH and SDHA. The primers were analyzed using the NetPrimer software tool site in order to avoid secondary structures, such as hairpins and loops. Primers crossed various exons to differentiate cDNA and genomic DNA amplification.

Real time PCR was carried out using LightCyclerTM (Roche Diagnostics, Mannheim, Germany) and SYBR Green I (Roche Diagnostics, Mannheim, Germany), according to manufacturer's instructions. Each sample was analyzed in a total reaction volume of 20 μL, consisting of 4 ng of cDNA, 1X SYBR Green I dye, the required amount of MgCl_2_, and forward and reverse primers (Table 1). Reactions were incubated in a LightCycler, following specific conditions for each gene (Table 1). Initial denaturation and denaturation steps were the same for all the primers, *i.e.* 95 °C for 5 min and 95 °C for 10 s, respectively. For each experiment, a non-template reaction was included as negative-control, besides duplicates of each sample. Threshold cycle (Ct) values were determined at the same fluorescence threshold line for all the genes, and the Ct value for each sample was obtained by calculating the arithmetic mean of duplicate values.

The specificity of RT-PCR products was evaluated by melting curve analysis, agarose gel electrophoresis and direct sequencing of amplified products. Melting curve analysis of the three genes was done within the range 70 °C to 95 °C at 0.1 °C/s. Sequencing was done using the DYEnamic ET DyeTerminator Sequencing Kit (Amersham Bioscience, Piscataway, NJ, USA) and the ABI 3100 Automated DNA Sequencer (Applied Biosystems).

Serial cDNA dilution curves were produced in order to calculate the amplification efficiency of all genes. A graph of threshold cycle (Ct) *vs.* log_10_ relative copy number of the sample from a dilution series was produced. The slope of the curve was used to determine amplification efficiency (Pfaffl, 2001): Efficiency = 10 ^(-1/slope)^.

To evaluate the stability of reference gene expression, the geNorm tool was applied (Vandesompele *et al.*, 2002). geNorm uses the average pair-wise variation of a gene in relation to the other genes analyzed, with the geometric mean as normalization factor. Genes are scored by a gene expression stability value (M-value). The gene with the highest M-value, *i.e.* the least stable, is then excluded in a stepwise fashion until the most stable genes are determined. Before analyses, Ct values were converted into relative quantities, with PCR efficiency in mind. Genes were analyzed both individually and group wise.

The General Linear Model (GLM) procedure of the Statistical Analysis System Institute (SAS, 1991) was applied for variance analysis of cycle threshold (Ct) values. Comparison of Ct means was carried out using a Student's t test. Results were expressed as arithmetic means (± standard deviation and coefficient of variation) of non-transformed data.

All RNA samples were examined for purity, integrity and concentration before cDNA synthesis. The absorbance ratios of all samples at OD_260_/OD_280_ were between 1.8 and 2.0, thereby indicating that all were free of proteins or phenol potentially accumulated during RNA extraction.

Following real-time PCR-specific conditions, the analysis of melting temperatures, amplicon sizes and sequencing data demonstrated the specificity of the PCR reactions. All genes presented a single peak in the melting curve, thereby indicating the absence of primer-dimer formation during the reaction and amplification specificity. The melting temperatures for all the genes are presented in Table 1. According to Zaros *et al.* (2007), melting curve analysis is a quick method for checking RT-PCR specificity. Nevertheless, in some cases, the melting temperatures of RT-PCR products of different lengths are the same, therefore indistinguishable by melting curve analysis. Consequently, amplified products were sequenced and gene identity was confirmed to guarantee specificity.

Efficiency values obtained for real-time PCR amplification of the three reference genes ranged from 1.92 to 1.98, this indicating that amplification efficiency was near the theoretical optimum level of 2 (Wilkening and Bader, 2004). Efficiency values (E), slope values and correlation coefficients (R^2^) for each primer pair are shown in Table 1.

Ct values for the three tissues ranged from 16.86 to 20.34 for RPLO, 22.77 to 28.06 for GAPDH and 25.71 to 28.40 for SDHA. The lower Ct value for RPLO indicated that this gene had reached the threshold of detection with less amplification cycles than GAPDH and SDHA, and thus was more abundant in the small intestine, abomasum lymph nodes and abomasum, respectively (Table 2). The coefficient of variation in the three tissues ranged from 2.13 to 5.53 for RPLO, 8.59 to 19.12 for GAPDH and 2.85 to 9.41 for SDHA, across all the tissues and groups. The mRNA levels for RPLO were not different (p > 0.05) between resistant and susceptible groups for each tissue. On the other hand, GAPDH and SDHA presented different mRNA levels in the abomasum tissue of resistant and susceptible groups (p < 0.05) (Table 2).

From geNorm analysis it was inferred that all the three genes reached high expression stability with low M values (Table 2) below the default limit of M = 1.5 (Vandesompele *et al.*, 2002). Furthermore, RPLO was ranked as the most stable gene, followed by SDHA in the abomasum and abomasal lymph nodes, in both resistant and susceptible animals. On the other hand and also in both groups, SDHA was selected as the best reference gene in the small intestine, followed by RPLO (Table 2). These differences in ranking reflect the existence of a tissue-specific metabolism and/or unknown tissue specific factors. The combination of RPLO and SDHA was suitable, yielding a V_2/3_ value of 0.015 for the resistant group and 0.010 for the susceptible in the abomasum, and 0.015 and 0.025 in lymph nodes, respectively. In the small intestine, the best combination was SDHA and RPLO, with V_2/3_ values of 0.015 and 0.038 for resistant and susceptible groups, respectively, all less than the cutoff value 0.15. The V value indicates systematic variation calculated as pair wise variation, for repeated RT-PCR experiments on the same gene.

When these reference genes were analyzed in all the samples together, *e.g.*, resistant and susceptible groups as one group, the same pattern of stability was observed.

Although GAPDH is widely used as an internal control gene and has traditionally been considered to be universal (Giulietti *et al.*, 2001; Bustin, 2002; Bustin *et al.*, 2005), problems associated with its use have been pointed out (Ke *et al.*, 2000; Suzuki *et al.*, 2000), as was confirmed in the present work. Zaros *et al.* (2007) evaluated GAPDH and RPL-19 expression in the abomasum, abomasal lymph nodes and the small intestine of cattle, both resistant and susceptible to gastrointestinal nematode infection, and ascertained that GAPDH is not suitable as a reference gene, because its expression was not constant across treatments and presented higher variation than RPL-19. Cao *et al.* (2006) also observed that GAPDH was not the most stable reference gene in studies with sheep. These data are in agreement with our findings in which GAPDH presented the greatest variation compared to ribosomal protein, the latter being the most stable gene of the two, in the abomasum and abomasal lymph nodes.

On the other hand, Garcia-Crespo *et al.* (2005) proved GAPDH to be the most stable gene in five out of six tissues in sheep susceptible to scrapie, with SDHA in second place, whereas RPL0 was considered to be a good reference gene in mesenteric lymph nodes. According to our results, RPL0 was found to be the most stable gene in the abomasum and abomasal lymph nodes, although less stable in the small intestine. Similarly, Bricarello *et al.* (2008) used RPL-19, a member of the ribosomal protein family, to determine cytokine gene expression (IL-2, IL-4, IL-8, IL-12p35, IL-13, TNF-a, IFN-g, MCP-1, MCP-2 and glycoprotein MUC-1) in Nelore cattle with different degrees of resistance to nematode infection, and obtained reliable results in its use as reference. Similar results were observed by Regitano *et al.* (2008), also with RPL-19 as reference gene, when studying mRNA levels of cytokines related to resistance in Nelore cattle, with and without *Boophilus microplus* infestation.

Although there are few reports on the use of SDHA as a reliable reference gene, our results suggest its usefulness as a reference gene for the small intestine from sheep infected by gastrointestinal nematodes. Similar results were obtained by Mulubamba *et al.* (2008) using SDHA as a reference gene in studies with sheep infected by *Mycobacterium avium*.

Accurate normalization of gene expression levels is an absolute prerequisite for reliable results, especially when biological significance of suitable gene expression differences is studied. Relatively little attention has been paid to the systematic study of normalization procedures and their impact on studies of gene expression.

Our findings clearly demonstrate that, without prior validation, the three reference genes used in sheep from temperate regions cannot be employed in studies involving tropical sheep. We further demonstrated that in Brazilian Somali sheep infected with endoparasites, there is no single universal reference gene for all the tissues. RPLO was ranked as the most appropriate reference gene for the abomasum and abomasal lymph nodule tissues, and SDHA as that for the small intestine. Consequently, this inherent variation has to be considered and the stability of the reference genes needs to be studied in each scenario prior to any relative quantification study in order to obtain results that are as accurate as possible.

## Figures and Tables

**Table 1:** PCR conditions and slope, correlation coefficient (R^2^) and efficiency values (E) for each reference gene.

Gene	Sequence	Amplicon (pb)	Primer (pMol)	Mg (mM)	Annealing (°C/s)	Extension (°C/s)	Melting (°C)	Slope	R^2^	E
RPLO	F: 5'CAACCCTGAAGTGCTTGACAT 3' R: 5'AGGCAGATGGATCAGCCA 3'	220	5	1.6	60/9	72/9	86.5	-3.349	0.997	1.988
GAPDH	F: 5' GGTGATGCTGGTGCTGAGTA 3' R: 5'TCATAAGTCCCTCCACGATG 3'	256	10	2.0	60/9	72/11	88.4	-3.515	0.956	1.927
SDHA	F: 5' ACCTGATGCTTTGTGCTCTGC 3' R: 5' CCTGGATGGGCTTGGAGTAA 3'	126	5	2.0	61/9	72/6	88.0	-3.413	0.995	1.964

**Table 2:** Analysis of RPLO, GAPDH and SDHA reference genes in the abomasum (AB), small intestine (SI) and abomasal lymph nodes (ALN) from resistant (R) and susceptible (S) groups.

Gene	Group	M value				Ct Mean				Standard deviation				Coeff. variation (%)		
		AB	SI	ALN		AB	SI	ALN		AB	SI	ALN		AB	SI	ALN
RPLO	R	0.035	0.050	0.045		20.34a	16.86a	18.11a		1.014	0.938	0.849		4.98	5.53	4.94
	S	0.025	0,056	0.048		20.06a	17.28a	18.23a		1.030	0.369	0.717		5.14	2.13	3.93
GAPDH	R	0.046	0.054	0.052		28.03a	23.38a	24.47a		2.60	2.99	3.27		9.283	12.80	13.36
	S	0.036	0.058	0.073		25.07b	24.63a	22.77a		2.15	4.71	4.20		8.596	19.12	18.46
SDHA	R	0.042	0.040	0.049		28.13a	25.71a	28.40a		0.801	1.90	2.61		2.85	7.41	9.22
	S	0.036	0.043	0.062		26.58b	25.95a	27.19a		1.41	2.44	2.04		5.33	9.41	7.53

Values with different letters in the same column are statistically different (p < 0.05).
